# Design, Synthesis and Pharmacological Evaluation of Novel 2-[2-(2-Chlorophenoxy) phenyl]-1,3,4-oxadiazole Derivatives as Benzodiazepine Receptor Agonists

**Published:** 2012

**Authors:** Mehrdad Faizi, Majid Sheikhha, Nematollah Ahangar, Hamed Tabatabaei Ghomi, Bijan Shafaghi, Abbas Shafiee, Seyyed Abbas Tabatabai

**Affiliations:** a *Department of Pharmacology and Toxicology, School of Pharmacy, Shahid Beheshti University of Medical Sciences, Tehran, Iran.*; b *Department of Medicinal Chemistry, School of Pharmacy, Shahid Beheshti University of Medical Sciences, Tehran, Iran.*; c *Department of Medicinal Chemistry, School of Pharmacy, Tehran University of Medical Sciences, Tehran, Iran.*

**Keywords:** Anticonvulsant, Benzodiazepine receptors, Synthesis, 1,3,4-Oxadiazoles, Conformational analysis

## Abstract

New derivatives of 2-[2-(2-Chlorophenoxy)phenyl]-1,3,4-oxadiazole as candidates for agonistic effect on benzodiazepine receptors were synthesized. Conformational analysis and superimposition of energy minima conformers of the novel compounds on estazolam, a known benzodiazepine agonist, revealed that the main proposed benzodiazepine pharmacophores were well matched. In pharmacological evaluation, anticonvulsant activity of the compounds determined by pentylenetetrazole-induced lethal convulsion and maximal electroshock tests. The results showed that the introduction of an amino substituent in position 5 of 1,3,4- oxadiazole ring generates compound 6 that has a considerable effect. Compound 8 with a hydroxyl substituent on position 5 of 1,3,4- oxadiazole ring showed a relatively mild anticonvulsant activity, which was significantly weaker than that of diazepam and compound 6. Anticonvulsant effects of active compounds were antagonized by flumazenil, an antagonist of benzodiazepine receptors, indicating the involvement of benzodiazepine receptors in these effects.

## Introduction

**Figure 1:**
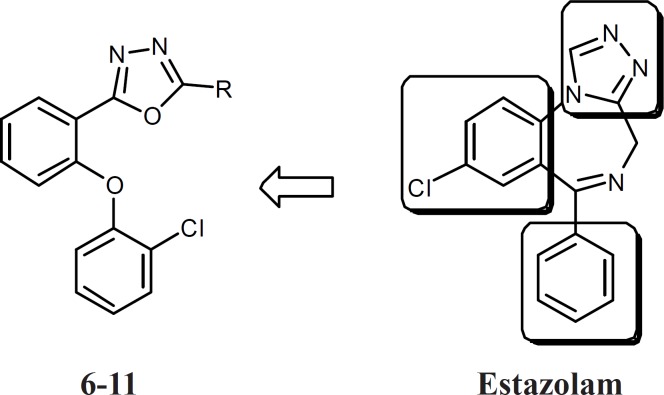
The structure of designed compounds 6-11 and estazolam. The main pharmacophores have been conserved

Agonists of benzodiazepine receptors are important classes of drugs used in the control of epilepsy, anxiety, muscle cramps, sleep problems and many other medical problems (1). Aside from their quick onset of action and low toxicity, benzodiazepines have some undesirable effects such as sedation, negative effect on cognition, and development of tolerance to the desirable effects (2). Therefore, synthesis of novel agonists of benzodiazepine receptors with different chemical structure is still an important challenge. Several models for structure-activity relationship of benzodiazepines have been suggested, but all of them have at least two features in common: One coplanar proton-accepting group, placed at a suitable distance from an aromatic ring. Another out-of-plane aromatic ring is also favorable for binding to the receptor (3-8). Based on these feutures and in continuance of our previous studies on five member heterocycle rings such as triazoles, oxadiazoles, and thiadiazoles, compounds 6-11 were designed which had all features of a benzodiazepine agonist (Figure 1), but had simpler and less rigid structures (9-17). Conformational analysis, followed by superimposition of energy minima conformers of estazolam and the novel compounds was performed to reveal whether the design compounds could mimic the benzodiazepine effects. As an in vivo evaluation of benzodiazepine effects, pentylenetetrazole (PTZ)-induced lethal convulsion and maximal electroshock (MES) tests were performed and the results for synthesized compounds were compared with diazepam.

## Experimental

*Chemistry*

A Kofler hot stage apparatus was used to obtain melting points. A Perkin–Elmer Model 550 SE spectrophotometer was used to obtain the UV spectra. A Nicolet FT-IR Magna 550 spectrophotometer was used to obtain the IR spectra. A Bruker FT-80 spectrometer was used to obtain the ^1^H NMR spectra and chemical shifts (*δ*) reported in ppm relative to internal tetramethylsilane. A Finnigan TSQ 70 Mass spectrophotometer at 70 eV was used to obtain mass spectra. All the chemicals for synthesis were supplied from Merck (Germany).

*2-(2-Chlorophenoxy)benzoic acid* *(*1*)*

9.7 g (421 mmol) of sodium was dissolved in dry methanol (250 mL) and 2-chlorophenol (28 g, 217 mmol) and of 2-chlorobenzoic acid (32 g, 204 mmol) were added. The solvent was distilled and dry dimethylformamide (100 mL) and a little amount of copper powder was added to the remaining salt. The mixture was heated at reflux for 2 h. The reaction was dark purple at the end. The reaction mixture was added to water (1 L) and while stirring, it was acidified with diluted hydrochloric acid. The resulting precipitates were filtered and washed with water and recrystalized in ethanol 79% to give 43 g (78%) of 1. mp:124-125°C; IR:(KBr) ν (cm^-1^) 3100-2500 (COOH), 1692(C=O); Mass m/z (%): 248 (M^+^, 100), 231 (90), 213 (21), 168 (30), 121 (83).

*Methyl 2-(2-chlorophenoxy)benzoate (* 2 *)*

25 g (100 mmol) of (1) was dissolved in methanol (400 mL) and concentrated sulfuric acid (8 mL) was added. The solution was heated at reflux for 7 h. Then, methanol was distilled and the remnant was alkalinized after being cooled in the ice bath with NaOH 20% and extracted with diethyl ether. The diethyl ether phase was washed first with aqueous NaOH 20% and then with water and was dried with anhydrous sodium sulfate and distilled to give 25 g (95%) of 2 as an oil. IR: (KBr) ν (cm^-1^) 1735 (C=O); 80MHz ^1^H-NMR(CDCl_3_): δ (ppm) 3.81 (s, 3H, COOCH_3_), 6.76-7.51 (m, 7H, aromatic), 7.94 (dd, 1H, J_5,6_= 7.6, J_4,6_= 2.0 Hz, phenyl H_6_); Mass m/z (%): 262 (M^+^, 65), 231 (100), 121 (100).

*2-(2-Chlorophenoxy) benzoic acid hydrazide (* 3 *)*

9.5 g (36 mmol) of 2 was dissolved in methanol (17 mL), hydrazine hydrate (9 mL, 180 mmol) was added, and stirred for 12 h at room temperature. Then, water (20 mL) was added and white precipitates was filtered, washed with water, and recrystallized with a mixture of ethanol and a few drops of water to give 8 g (84%) of 3. mp: 122-124°C; IR: (KBr) ν (cm^-1^) 3315, 3203 (NH2), 1630 (C=O); 80 MHz ^1^H-NMR (CDCl3): δ (ppm) 4.18 (broad s, 2H, NH2), 6.66 (dd, 1H, J_5,6_=8.8, J_4,6_=1.6 Hz, phenoxy H_6_), 7.00-7.57(m, 6H, aromatic), 8.23 (dd, 1H, J_5,6_= 7.2, J_4,6_= 2.5 Hz, phenyl H_6_), 8.50 (broad s, 1H, NH); Mass m/z (%): 262 (M^+^, 52), 231 (100), 121 (100), 111 (24).

*1-[2-(2-Chlorophenoxy)benzoyl]-2-formylhydrazine (* 4 *)*

A solution of 3 (2.5 g, 9.5 mmol) in formic acid (25 mL) was heated at reflux for 4 h. The solution was cooled and water was added. The precipitate was washed with water and recrystallized in ethanol to give 2.25 g (81%) of 4. mp: 138-139°C; IR: (KBr) ν (cm^-1^) 3360, 3349 (NH), 1661,1643 (C=O); 80 MHz ^1^H-NMR (CDCl_3_): δ (ppm) 6.65 (d, 1H, J_4,6_= 7.8 Hz, phenoxy H_6_), 7.26-7.46 (m, 6H, aromatic), 8.22 (s, 1H, COH), 8.23-8.30 (m, 1H, phenyl H_6_), 9.37 (broad s, 1H, NH), 10.29 (broad s, 1H, NH); Mass m/z (%): 290 (M^+^, 52), 231 (100).

*2-[2-(2-Chlorphenoxy)phenyl]-1,3,4-oxadiazole (* 5 *)*

3g (10.3 mmol) of 4 was dissolved in dry xylene (90 mL), P_2_O_5_ (3 g) was added to the solution, and the mixture was heated at reflux for 3 h. Then, xylene was decanted and the precipitate was washed with xylene. The xylene phase was distilled and the remnant was purified by thin layer chromatography using a solvent system composed of chloroform and methanol (98:2) to give 1.69 g (60%) of 5 as an oily product. IR: (KBr) ν (cm^-1^) 3130 (C=C aromatic), 1671 (C=N); 80 MHz ^1^H-NMR (CDCl_3_): δ (ppm) 6.79-7.58(m, 7H, aromatic), 8.14 (dd, 1H, J_5,6_= 8.0, J_4,6_= 1.6 Hz, phenyl H_6_), 8.48 (s, 1H, oxadiazole); Mass m/z (%): 272 (M^+^, 2.5), 237 (100), 182 (35), 152 (12), 139 (10), 77 (2.5), 51.5 (5).

*2-amino-5-[2-(2-chlorophenoxy)phenyl]-1,3,4-oxadiazol (* 6 *)*

A solution of sodium bicarbonate (2.17 g, 25.8 mmol) in water (40 mL) was added to a solution of 3 (6.8 g, 25.9 mmol) in dioxane (60 mL) and the mixture was stirred for 5 min at room temperature. Then, cyanogen bromide (2.75 g, 26 mmol) was added and the reaction mixture was stirred at room temperature overnight. Then, water was added and a white mass precipitated. The precipitate was recrystallized in ethanol to give 6 g (80%) of 6, mp: 144-146°C, IR: (KBr) ν (cm^-1^) 3335, 3124 (NH2), 80 MHz ^1^H-NMR (CDCl_3_): δ (ppm) 5.54 (broad s, 2H, NH2), 6.78-7.50 (m, 7H, aromatic) 7.98 (dd,1H, J_5,6_= 7.3, J_4,6_= 2.1 Hz, phenyl H_6_). Mass (m/z, %): 287 (M^+^, 6), 252 (100), 209 (9),182 (25),152 (27).

*5-[2-(2-Chlorophenoxy)phenyl]-1,3,4-oxadiazole-3(H)-2-thione (* 7 *)*

Carbon disulfide (6.4 mL) was added drop wise to a solution of 3 (8 g, 30.4 mmol) and KOH (1.87 g, 33 mmol) in ethanol 96% (300 mL) in ice bath and the reaction mixture was heated at reflux for 7 h. Then, the solvent was distilled and water was added to the remnant and the mixture was acidified with hydrochloric acid (1 N). The precipitate was filtered and washed with water and recrystallized to give 9 g (97%) of **7**. mp: 195-197°C, IR: (KBr) ν (cm^-1^) 3197 (NH); 80 MHz ^1^H-NMR (CDCl_3_): δ (ppm) 6.80-7.51 (m, 7H, aromatic), 8.10(dd, 1H, J_5,6_=7.1, J_4,6_=2.1 Hz, phenyl H_6_); Mass (m/z, %): 304 (M^+^, 64), 269 (12), 209 (46), 181 (100), 152 (66) 121 (65), 77 (12).

*5-[2-(2-Chlorophenoxy)phenyl]-1,3,4-oxadiazole-3(H)-2-one (* 8 *)*

To a mixture of 3 (4.4 g, 15.2 mmol) and triethylamine (2.4 mL, 15.2 mmol) in THF (150 mL) at 0°C, 1,1-carbonyldiimidazole (3.68 g, 22.7 mmol) was added and the mixture was stirred for 5 h at 0°C. Then, triethylamine (1.6 mL) and 1,1-carbonyldiimidazole (2 g) was added and the mixture was stirred overnight at room temperature. The solvent was distilled and the remnant was dissolved in diethyl ether and washed with hydrochloric acid (1 N), saturated sodium bicarbonate, and saturated NaCl solution respectively. The diethyl ether phase was dried with anhydrous sodium sulfate and distilled. The residue was recrystallized in ethanol 96% to give 3.7 g (84%) of 8. mp: 178-180°C, IR: (KBr) ν (cm^-1^) 3078, (NH), 1782(C=O); 80 MHz ^1^H-NMR (CDCl_3_): δ (ppm) 6.85-7.54 (m, 7H, aromatic), 8.12(dd, 1H, J_5,6_=7.0, J_4,6_= 2.2 Hz, phenyl H_6_); Mass (m/z, %): 288 (M^+^, 36), 253 (100), 209 (34), 182 (67), 139 (27), 77 (12), 51 (17).

*2-[(2-(2-chlorophenoxy)phenyl]-5-* *phenylamino-1,3,4-oxadiazole* *(*9*)*

Phenylisocyanate (270 mg, 2 mmol) was added to a solution of 3 (525 mg, 2 mmol) in dry toluene or benzene(10 ml) and heated at reflux for 30 min. Then, N,N΄-dicyclohexylcarbodiimide (618 mg, 3 mmol) was added and heating at reflux was continued for 7 h. The reaction mixture was cooled, filtered, and the precipitate washed with cool toluene. The result was separated by thin layer chromatography using a solvent system of hexane-ethyl acetate (1:1) and then recrystallized in methanol plus a few drops of water to give 300 mg (41%) of 9. mp: 140-142°C, IR: (KBr) ν (cm^-1^) 3150 (NH); 80 MHz ^1^H-NMR (CDCl_3_): δ (ppm) 6.62-7.56 (m, 12H, aromatic), 8.1 (dd, 1H, J_5,6_=7.3, J_4,6_= 2.0 Hz, phenyl H_6_), 8.32 (broad s, 1H, NH). Mass (m/z, %): 363 (M^+^, 9), 328 (100), 182 (23), 92 (13), 77 (16). 

*2-[2-(2-Chlorophenoxy)phenyl]-5-methylthio-1,3,4-oxadiazole (* 10 *)*

To a solution of 8 (1.5g, 4.92 mmol) in ethanol 96% (1.3 mL) in a vial, methyl iodide (0.71 g, 5 mmol) and NaOH 10% (2 mL) were added. The vial was put into an ultrasonicator for 5 min. Then, the reaction mixture was poured into water and stirred until a pure white compound precipitated. The precipitate was filtered and washed with water to give 1.3 g (82%) of 10. mp: 58-60°C, IR: (KBr) ν (cm^-1^) 1577,1471 (C=C, aromatic); 80 MHz ^1^H-NMR (CDCl_3_); δ (ppm) 2.69 (s, 3H, SCH3), 6.82-7.53 (m, 7H, aromatic), 8.07(dd, 1H, J_5,6_= 7.2, J_4,6_= 2.3 Hz, phenyl H_6_); Mass (m/z, %): 318 (M^+^, 5), 283 (100), 182 (40), 103 (35).

*2-Benzylthio-5-[2-(2-chlorophenoxy)phenyl]-1,3,4-oxadiazole* *(*11*)*

To a solution of 8 (1.5g, 4.92 mmol) in ethanol 96% (1.3 mL) in a vial, benzyl chloride (0.62 g, 4.92 mmol) and NaOH 10% (2 mL) were added. The vial was put into an ultrasonicator for 5 min. Then, the reaction mixture was poured into water and stirred until a pure white compound precipitated. The precipitate was filtered and washed with water to give 1.34 g (69%) of 11. mp: 56-58 °C; IR: (KBr) ν (cm^-1^) 1573,1461 (C=C, aromatic); 80 MHz ^1^H-NMR (CDCl_3_): δ (ppm) 4.44 (S, 2H, CH2), 6.80-7.53 (m,12H, aromatic), 8.07(dd, 1H, J_5,6_= 7.5, J_4,6_= 2.0 Hz, phenyl H_6_); Mass (m/z, %): 316 (30), 191 (20), 147 (15).

*Conformational analysis*

Conformational analysis of the novel compounds and estazolam were performed by MMX force field method followed AM1 calculation, implemented in Hyperchem software (Hypercube Inc.). Global energy minima conformers of the designed compounds were superimposed on corresponding conformer of estazolam molecule which was considered as a reference benzodiazepine agonist.

*Pharmacological evaluation*

Male NMRI mice (Pasteur Institute, Iran) weighting in the range of 20–25 g were used in the experiments. The animals were housed in a temperature controlled condition and 12 h light/dark cycle. All pharmacological experiments were performed between 9:00 and 15:00. Standard mouse diet and water were freely available for them except during the experiment. Thirty minutes before the experiment, the animals were selected randomly and transferred into individual cages and allowed to acclimatize before injection of drugs or vehicle. The novel compounds, flumazenil (Sigma), and diazepam (Sigma) were given IP as freshly prepared solutions. The novel compounds were dissolved in a mixture of 50% DMSO and 50% water. PTZ was dissolved in water and diazepam and flumazenil was dissolved in pure DMSO. If the solvent was mixture of DMSO and water, the injection volume was 10 mL/kg and if the solvent was only DMSO, the injection volume was 5 ml/kg, in order to avoid unwanted CNS depressant effects of DMSO. This study was conducted in accordance with protocols approved by the Institutional animal care and use committee and all experiments were performed based on the National Institutes of Health (NIH) Guide for the Care and Use of Laboratory Animals and all efforts were made to minimize the number of animals used in the study.

*Pentylenetetrazole model*

The novel compounds, vehicle, or diazepam were injected 30 min and flumazenil 15 min before the injection of PTZ. Then PTZ (100 mg/kg; IP) was injected. Mice were under observation for 30 min after the injection of PTZ and the dead mice were counted.

*Maximal electroshock model*

The novel compounds, vehicle, or diazepam were injected 30 min and flumazenil 15 min before the induction of seizure. The electrodes were connected to the ears of the mice and an alternative electric current (60 Hz, 50 mA) was connected to the animals for 0.2 sec. The mice were observed for 30 sec for occurrence of hind limb tonic extension (HLTE). 

**Figure 2:**
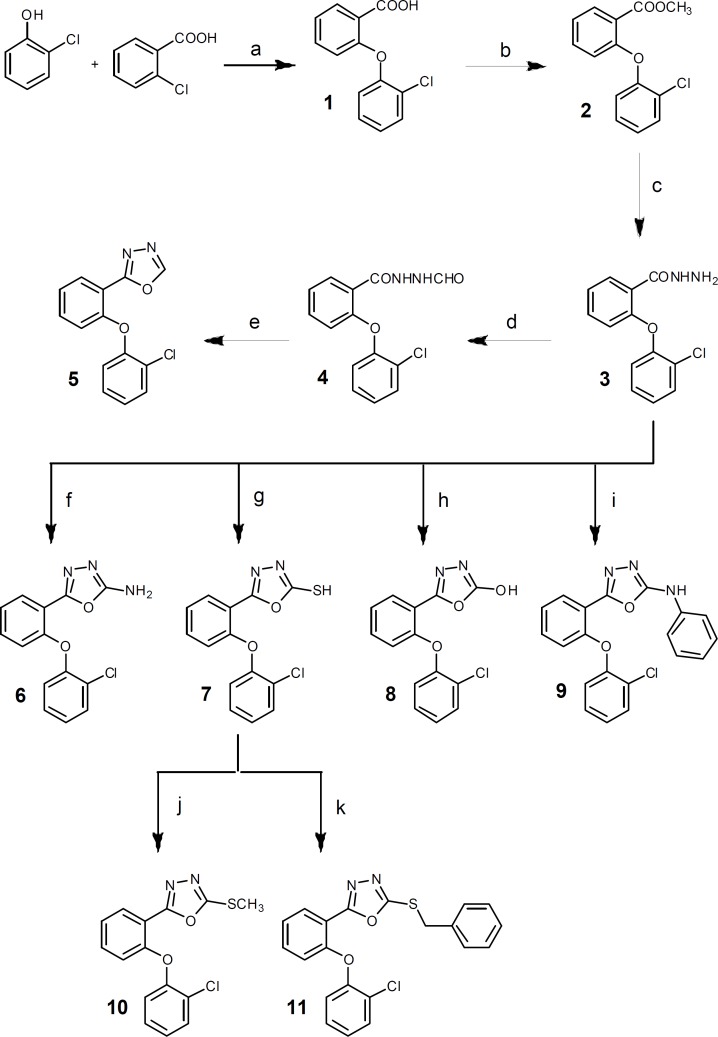
Reagents: (a) 1) Na, dry methanol, rt, 2h; 2) DMF, Cu, reflux; (b) methanol, H2SO4, reflux, 7 h; (c) methanol, NH2NH2.H2O, stir, rt, 12 h; (d) formic acid, reflux, 4 h; (e) P2O5, xylene, reflux, 3 h; (f) BrCN, dioxan, stir, rt, overnight; (g) CS2, ethanol, KOH, reflux, 7 h; (h) 1,1΄-carbonyldiimidazole, triethylamine, dry THF, 0°C, 5h then rt, overnight; (i) toluene, phenylisocyanate, DCC, reflux, 7 h; (j) methyl iodide, ethanol, NaOH, ultrasonication, 5 min; (k) benzyl chloride, ethanol, NaOH, ultrasonication, 5 min

*Statistical analysis*

Probit-regression method and SPSS software (Chicago, IL; version 13) were used to determine ED_50_. Fisher’s exact probability test was used to analyze the difference between the ED_50_ of the novel compounds in experimental groups. All the data were presented as Mean (95% confidence limits) and p < 0.05 considered statistically significant.

## Results

*Chemistry*

The designed compounds were synthesized according to Figure 2. 2-(2-Chlorophenoxy)benzoic acid 1 was synthesized through a nucleophilic aromatic substitution reaction of 2-chlorobenzoic acid and 2-chlorophenol (14, 18). Following esterification of 1, 2-(2-Chlorophenoxy) benzoic acid hydrazide 3, the key intermediate of the synthesis, was prepared by the reaction of corresponding ester 2 with hydrazine hydrate (19). Refluxing 3 in formic acid followed by heating the resulting formilated compound 4 in xylene with P_2_O_5_ provided 5 in good yield (20). Treatment of 3 with cyanogen bromide, carbon disulfide, 1,1´cabonyldiimidazole, phenylisocyanate in appropriate conditions gave 6, 7, 8 and 9 respectively (19-22).

**Figure 3:**
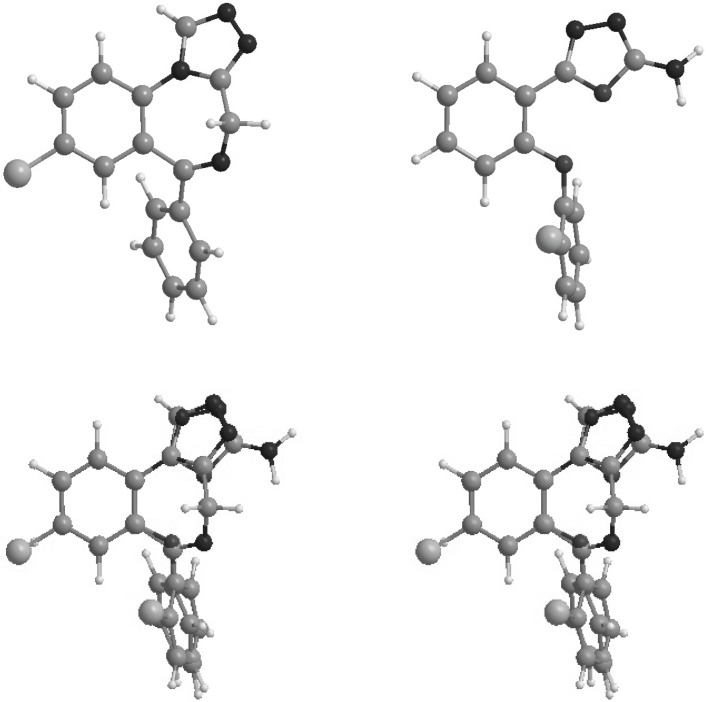
Stereoview of the superimposition of the energy minima conformers of estazolam (top left) and compound 6 (top right).

*Conformational analysis*

Conformations of the synthesized molecules and estazolam, a known benzodiazepine agonist, were energetically minimized using AM1 calculations. Figure 3 shows superimposition of compound 1**,** the most potent synthesized analogue, and estazolam. It is clear that aromatic rings, and proton accepting groups, the nitrogen atoms in position 3 of the oxadiazole and position 2 of the imidazole rings, are well matched.

*Pharmacological evaluation*

Benzodiazepine agonistic activity of the synthesized compounds and diazepam, as a reference, were tested using two well-known models: MES and PTZ (Table 1). Compounds 6 and 8 had significant anticonvulsant effects and compound 6 was the most potent. Significant reduction of the activity of the compounds by flumazenil indicates benzodiazepine agonistic effects for them.

## Discussion

The anticonvulsant activity of compounds 6-11 is shown in Table 1. Diazepam was considered as a standard benzodiazepine agonist. The results show that compound 6 with an amino substituent on 2 position of 1,3,4-oxadiazole ring has a considerable anticonvulsant activity and replacement of this group with OH (compound 8), decreases the anticonvulsant effect. The other groups decrease the effect under the considered acceptable limit (ED_50_ > 100 mg/kg). Since the activity of compounds 6 and 8 as well as diazepam has been significantly reduced by flumazenil, we can conclude that this effect is mediated through benzodiazepine receptors. These results are completely compatible with our previous studies on the other 1,3,4-oxadiazole and I,2,4-triazole derivatives and in all previous heterocycles which we have introduced as benzodiazepine receptor ligands, the amino substituent at the same position had the best effect (9-17). 

**Table 1:** Pharmacological evaluation of synthesized 1,3,4-oxadiazoles

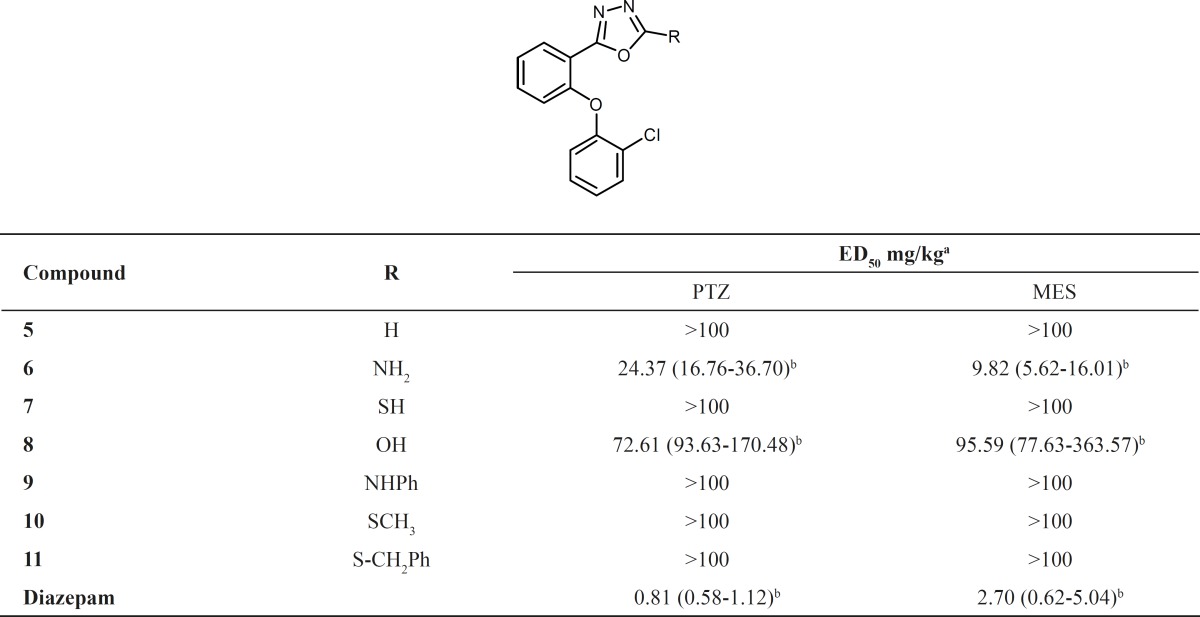

^a^n = 6, 95% confidence limits in parenthesis, LD_50_ of all compounds > 300 mg/kg.

^ b^ED_50 _ was significantly increased in the presence of flumazenil 10 mg/kg (p < 0.05).

Figure 3 illustrates the superimposition of energy minima conformers of compound 6 on estazolam as a reference benzodiazepine agonist. It is clear that the aromatic rings and proton accepting groups, N-2 on estazolam and N-3 on compound 6**, **are well matched. These groups could be considered as the main proposed benzodiazepine pharmacophores. Therefore, it is not surprising that compound 6 could mimic the benzodiazepine structure at the receptor sites. Although compound 6 is weaker than diazepam, this simple non-rigid structure could be a valuable lead compound for further researches. The use of high dose of PTZ might lead to fail to notice the occurrence of some early stages of the seizure behavior in animals associated with petit mal seizure in human. Therefore, some of the novel compounds might have significant anti seizure activity in other models using low doses of PTZ.
